# Personal

**Published:** 1916-05

**Authors:** 


### PERSONAL.

---

**Announcement of removal, travel, and other matters of interest are requested. Please report errors in the listing of any physician in the State and other directories, that we may co-operate with the State Society in securing a correct list.**

---

Dr. C. R. Calkins of Perry returned from a trip to Florida the last of March.

---

Dr. A. Crandall Way of Perry left for Florida April 8.

---

Dr. Chas. A. Richards has resigned from the Dunkirk Board of Health.

---

Dr. Geo. E. Fell of Buffalo has moved to 209 Porter Ave.

---

Dr. J. Henry Dowd of Buffalo has moved to 9 North Pearl Street.

---

Dr. Clarence Arthur McWilliams of N. Y., late Chief Surgeon of Military Hospital No. 32 at Chateau de Passy, France, gave a talk, illustrated with lantern slides, at the University Club of Buffalo, April 22, on The War at the French Front.

---

Dr. F. Park Lewis of Buffalo has been appointed a vice-president of the B. F. D. Permanent Blind Relief War Fund, whose headquarters are at 590 Fifth Avenue, N. Y.

---

The office of Dr. Frank A. Beyer, over 559 Main Street, Buffalo, was robbed April 2, at night.

---

Dr. John H. Pryor of Buffalo has been appointed Medical Adviser and Chief of the Consulting Staff of the J. N. Adam Memorial Hospital.

---

Dr. B. F. Rogers of Buffalo returned about April 1 from a western trip.

---

Dr. Maud A. Frye of Buffalo returned from Washington April 10.

---

Dr. Juliet E. Hanchett has resigned the position of public vaccinator of Syracuse and has sailed for Europe. Her Syracuse service extended over 15 years and included over 30,000 vaccinations of school children, without a bad result.

---

Dr. Roy S. Moore of Cicero has sold his practice to Dr.

F. B. Foote and has gone to Harvard for a post graduate course in eye, ear, nose and throat.

Dr. Floyd M. Crandall has been appointed by the Council of the State Society to succeed the late Dr. Wisner R. Townsend as Secretary.

Dr. S. P. Jewett of Buffalo has moved to Astoria, L. I.

Dr. Edward H. Storck of Buffalo was married to Miss Jane R. Henn March 29.

The home of Dr. C. H. Cook, 98 Rodney Avenue, Buffalo, was badly damaged by fire due to an electric iron last month.

Dr. Raymond Hensel and Dr. John T. Gratz (Buffalo 1914) have been formally appointed resident physicians in the municipal hospital.

Dr. Ulysses B. Stein, formerly of Buffalo, is located at Newfane.

Dr. F. C. Storer, formerly of Holly, is located at 296 Rugby Avenue, Rochester.

The Medical Advisory Board of Niagara Falls, on March 3, presented a loving cup to Dr. Wm. H. Hodge, who organized the Board in 1905 and has served on it ever since, several years as chairman.

A complimentary dinner was given to Health Commissioner Francis E. Fronczak of Buffalo at the Statler March 15, over 100 being present. Dr. F. Park Lewis presided. In responding to Dr. Lewis's introduction, Dr. Fronczak paid tributes to his predecessors: G. H. Mackey, Health Officer 1878-9, now Medical Director of the Equitable Life Insurance Co. in N. Y.; Lt.-Col. Albert H. Briggs, M. D., 1880-1 and 1884-7; Dr. Wm. C. Phelps, 1882-3; Dr. Edward Clark, 1888-9; Dr. Walter D. Greene, 1890-1 and 1902-6; Dr. Ernest Wende, the first to hold the title of Health Commissioner under the revised charter 1892-1901, and also 1907 till his death, Feb. 11, 1910, when Dr. Fronczak, who had been appointed Assistant Health Commissioner in 1905, became Commissioner, and has since been twice reappointed to the same office. Dr. John C. McEvitt, Lieutenant Commander and Surgeon in the State Naval Militia, responded to a toast on Surgical Preparedness, outlining the probable number of surgeons who would be called out in the various lines of defense. Dr. Linsly R. Williams, Deputy State Health Commissioner, spoke particularly of the part played by Buffalo in Public Health matters. Dr. Otto Pfaff, Mayor of Oneida, emphasized the duty of the profession in backing up and participating in the government of the state.

---

Dr. James D. V. Sheehan has been appointed Vaccinator of Syracuse.

---

Drs. Daniel F. Luby, John Van Duyn and Edward S. Van Duyn, of Syracuse, have left for Europe, to serve in the Auxiliary Hospital at Dieppe.

---

Dr. Frederick J. Parmenter has returned to Buffalo after having completed the course of study at the Post-Graduate Hospital in New York. Dr. Parmenter has relinquished his medical practice, and will confine his work in the future to surgery, paying special attention to the Cystoscopic Diagnosis and the major surgery of the Genito-Urinary system.

---

Dr. Thomas E. Soules of Buffalo left the middle of April for a month's trip down the Mississippi and to New Orleans and vicinity.

